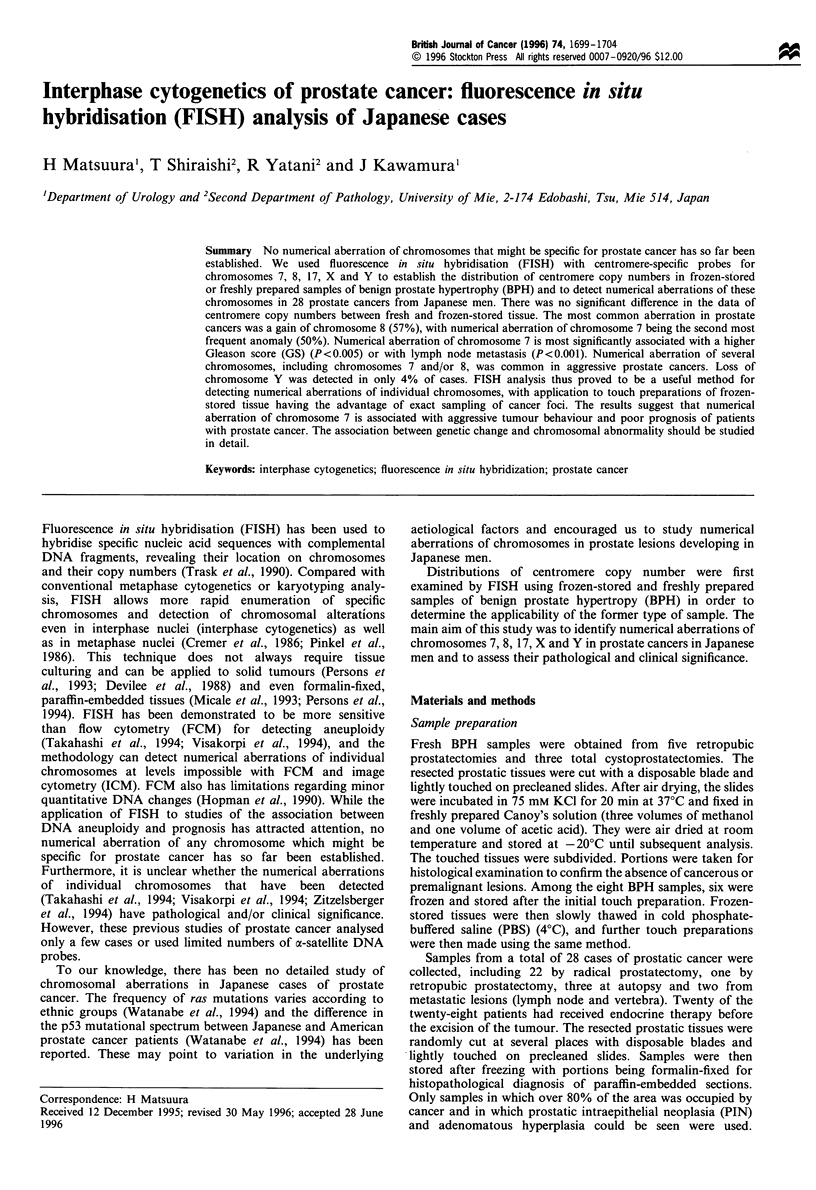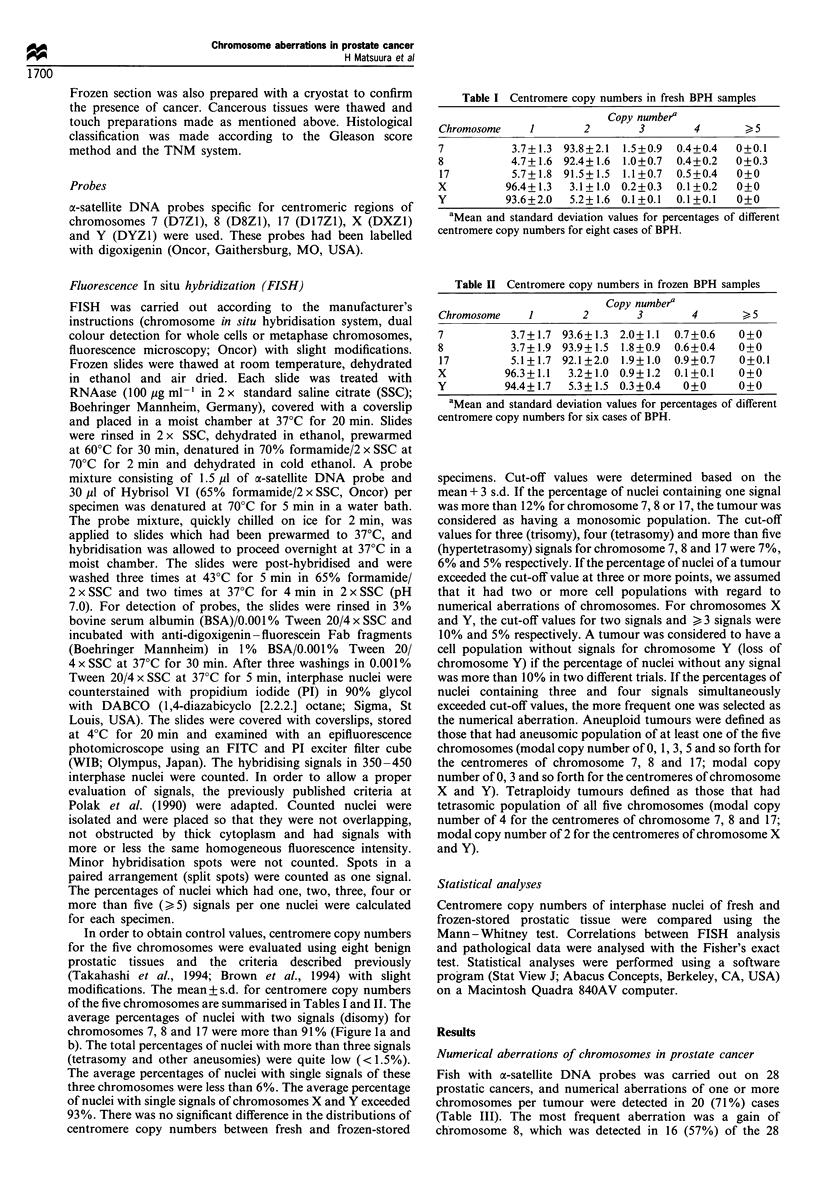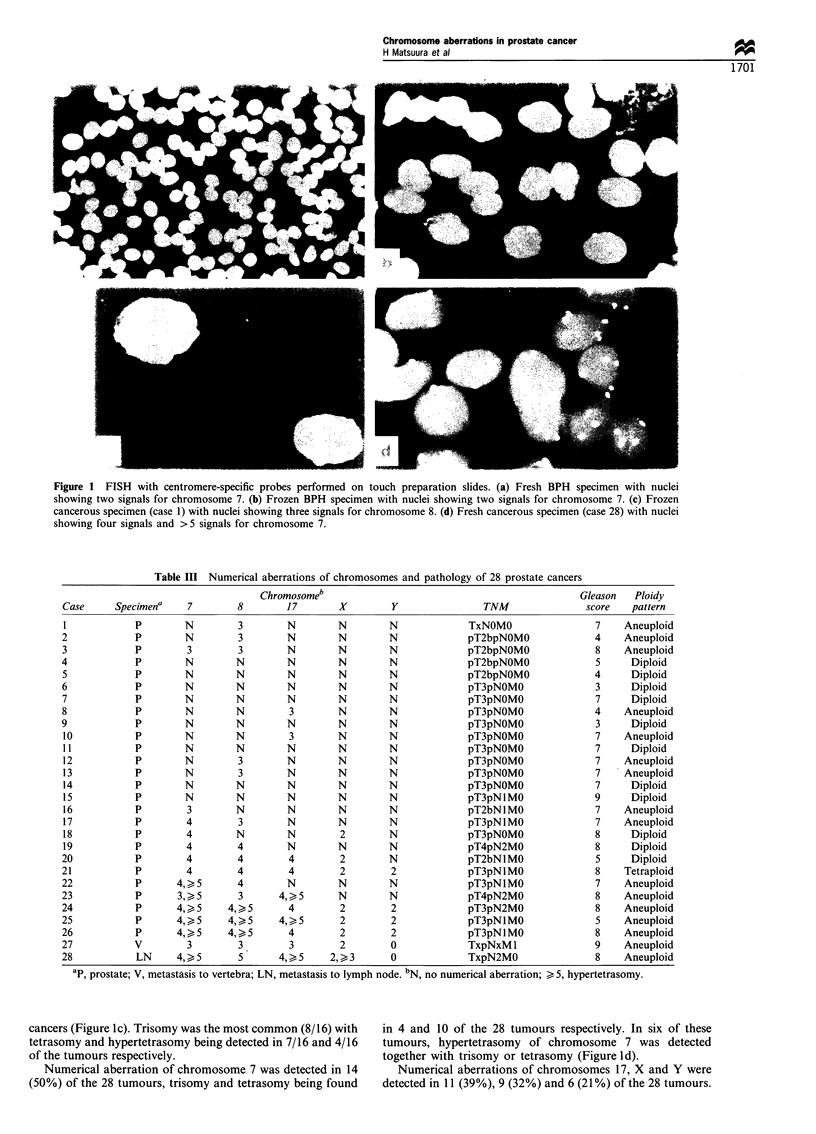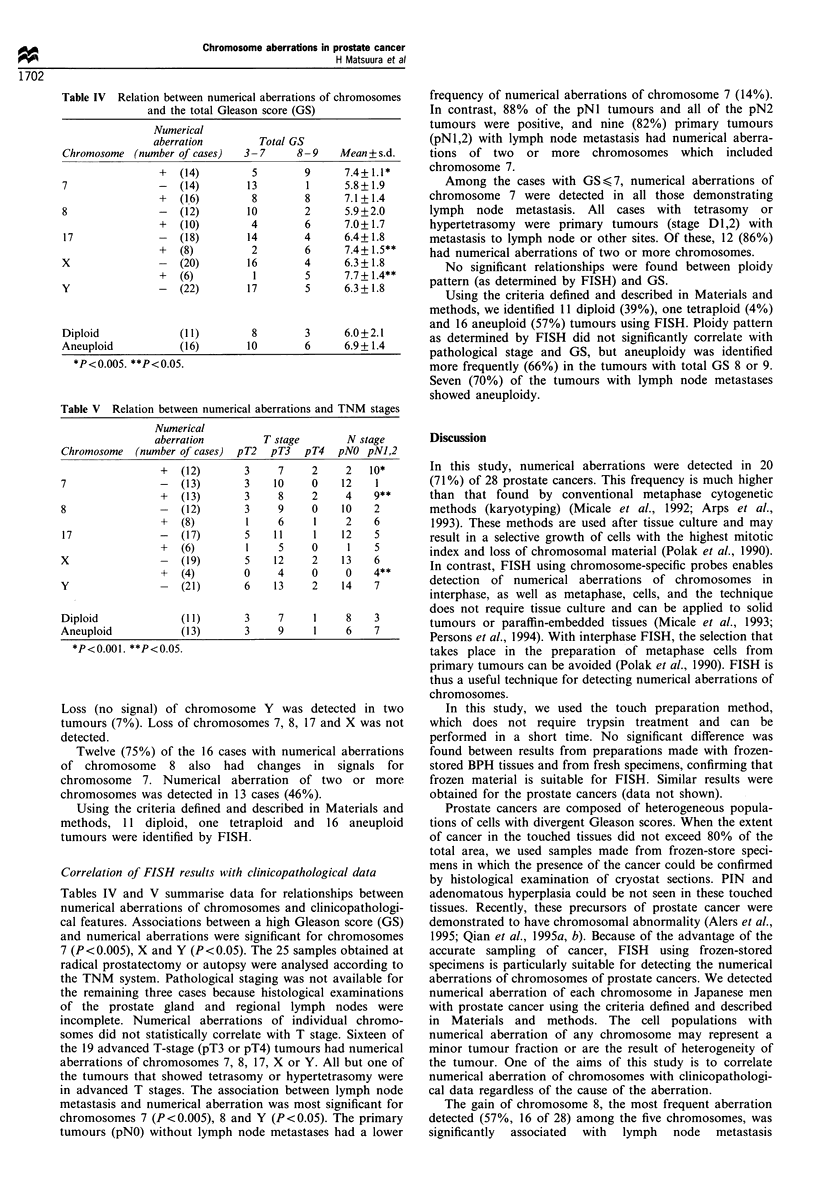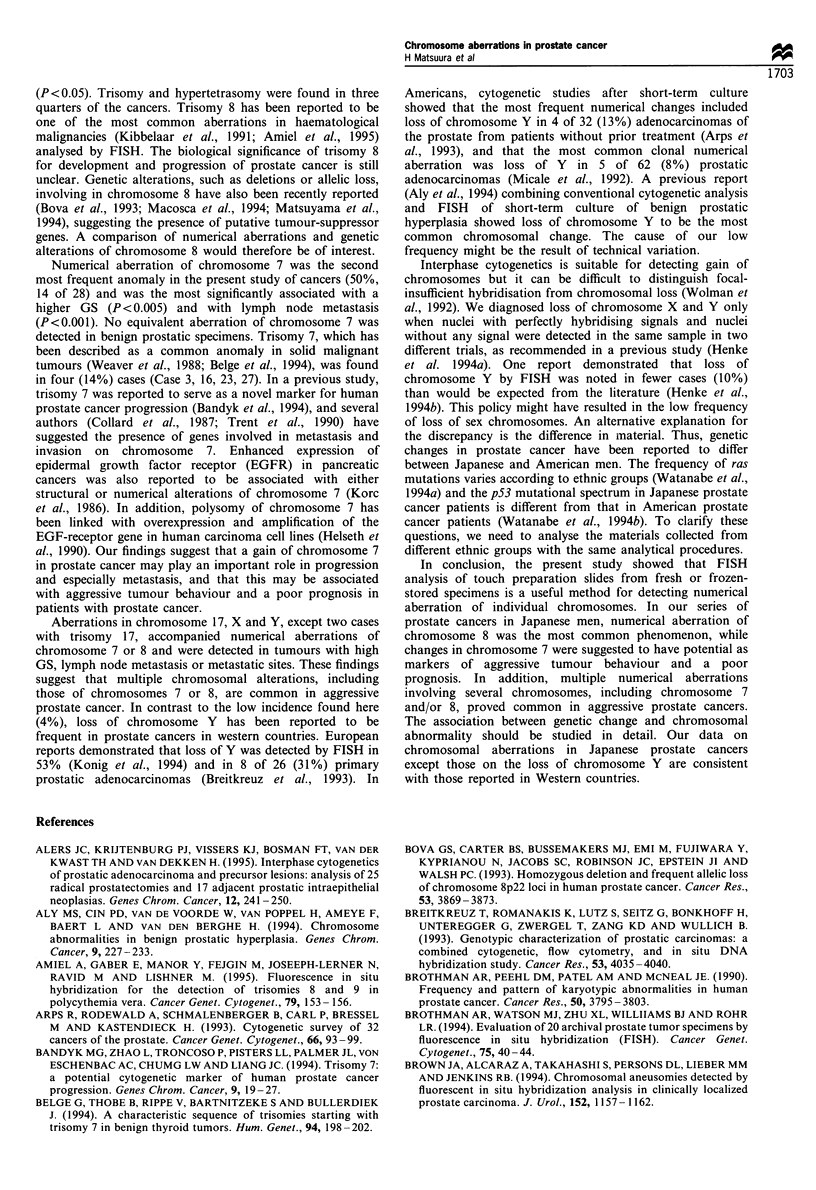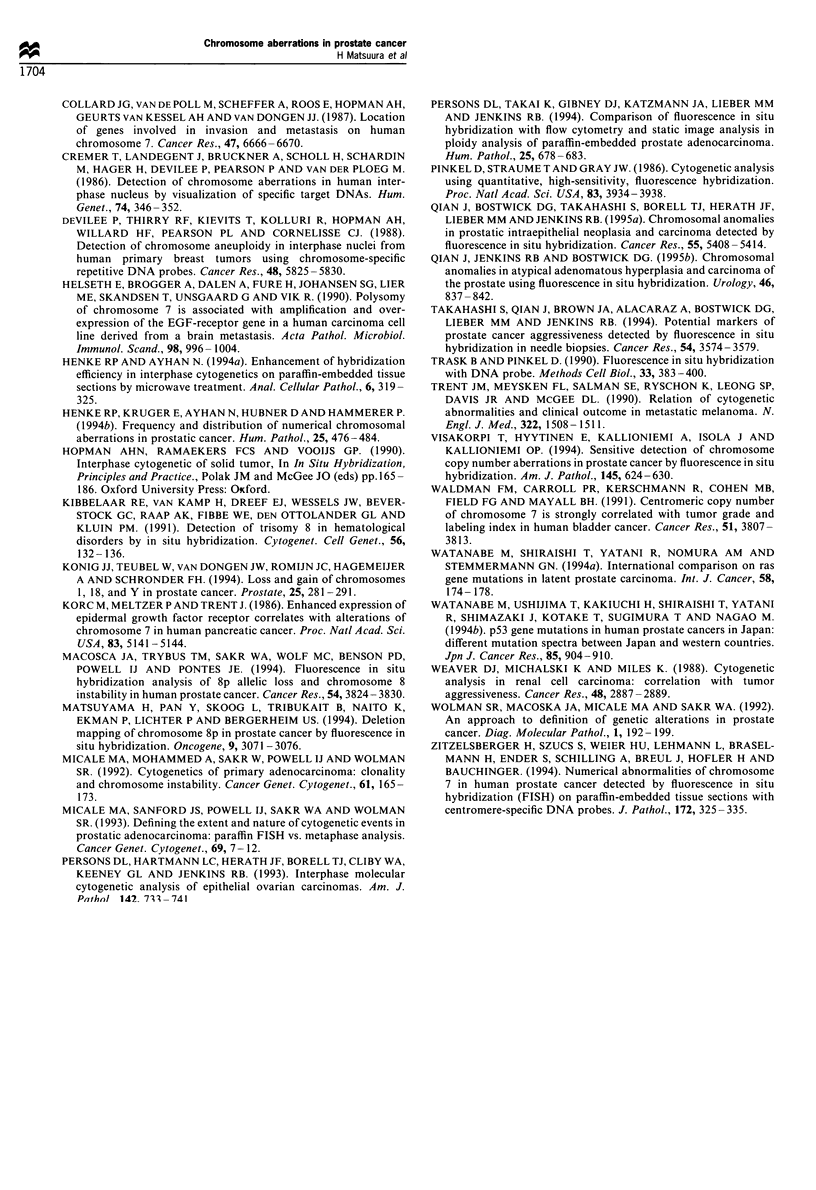# Interphase cytogenetics of prostate cancer: fluorescence in situ hybridisation (FISH) analysis of Japanese cases.

**DOI:** 10.1038/bjc.1996.617

**Published:** 1996-12

**Authors:** H. Matsuura, T. Shiraishi, R. Yatani, J. Kawamura

**Affiliations:** Department of Urology, University of Mie, Japan.

## Abstract

**Images:**


British Journal of Cancer (1996) 74, 1699-1704
© 1996 Stockton Press All rights reserved 0007-0920/96 $12.00

### Interphase cytogenetics of prostate cancer: fluorescence *in situ* hybridisation (FISH) analysis of Japanese cases

H Matsuura[1], T Shiraishi[2], R Yatani[2] and J Kawamura[1]

[1]*Department of Urology and* [2]*Second Department of Pathology, University of Mie, 2-174 Edobashi, Tsu, Mie 514, Japan*

**Summary** No numerical aberration of chromosomes that might be specific for prostate cancer has so far been established. We used fluorescence *in situ* hybridisation (FISH) with centromere-specific probes for chromosomes 7, 8, 17, X and Y to establish the distribution of centromere copy numbers in frozen-stored or freshly prepared samples of benign prostate hypertrophy (BPH) and to detect numerical aberrations of these chromosomes in 28 prostate cancers from Japanese men. There was no significant difference in the data of centromere copy numbers between fresh and frozen-stored tissue. The most common aberration in prostate cancers was a gain of chromosome 8 (57%), with numerical aberration of chromosome 7 being the second most frequent anomaly (50%). Numerical aberration of chromosome 7 is most significantly associated with a higher Gleason score (GS) ($P<0.005$) or with lymph node metastasis ($P<0.001$). Numerical aberration of several chromosomes, including chromosomes 7 and/or 8, was common in aggressive prostate cancers. Loss of chromosome Y was detected in only 4% of cases. FISH analysis thus proved to be a useful method for detecting numerical aberrations of individual chromosomes, with application to touch preparations of frozen-stored tissue having the advantage of exact sampling of cancer foci. The results suggest that numerical aberration of chromosome 7 is associated with aggressive tumour behaviour and poor prognosis of patients with prostate cancer. The association between genetic change and chromosomal abnormality should be studied in detail.

**Keywords:** interphase cytogenetics; fluorescence *in situ* hybridization; prostate cancer

Fluorescence *in situ* hybridisation (FISH) has been used to hybridise specific nucleic acid sequences with complemental DNA fragments, revealing their location on chromosomes and their copy numbers (Trask *et al.*, 1990). Compared with conventional metaphase cytogenetics or karyotyping analysis, FISH allows more rapid enumeration of specific chromosomes and detection of chromosomal alterations even in interphase nuclei (interphase cytogenetics) as well as in metaphase nuclei (Cremer *et al.*, 1986; Pinkel *et al.*, 1986). This technique does not always require tissue culturing and can be applied to solid tumours (Persons *et al.*, 1993; Devilee *et al.*, 1988) and even formalin-fixed, paraffin-embedded tissues (Micale *et al.*, 1993; Persons *et al.*, 1994). FISH has been demonstrated to be more sensitive than flow cytometry (FCM) for detecting aneuploidy (Takahashi *et al.*, 1994; Visakorpi *et al.*, 1994), and the methodology can detect numerical aberrations of individual chromosomes at levels impossible with FCM and image cytometry (ICM). FCM also has limitations regarding minor quantitative DNA changes (Hopman *et al.*, 1990). While the application of FISH to studies of the association between DNA aneuploidy and prognosis has attracted attention, no numerical aberration of any chromosome which might be specific for prostate cancer has so far been established. Furthermore, it is unclear whether the numerical aberrations of individual chromosomes that have been detected (Takahashi *et al.*, 1994; Visakorpi *et al.*, 1994; Zitzelsberger *et al.*, 1994) have pathological and/or clinical significance. However, these previous studies of prostate cancer analysed only a few cases or used limited numbers of α-satellite DNA probes.

To our knowledge, there has been no detailed study of chromosomal aberrations in Japanese cases of prostate cancer. The frequency of *ras* mutations varies according to ethnic groups (Watanabe *et al.*, 1994) and the difference in the p53 mutational spectrum between Japanese and American prostate cancer patients (Watanabe *et al.*, 1994) has been reported. These may point to variation in the underlying aetiological factors and encouraged us to study numerical aberrations of chromosomes in prostate lesions developing in Japanese men.

Distributions of centromere copy number were first examined by FISH using frozen-stored and freshly prepared samples of benign prostate hypertropy (BPH) in order to determine the applicability of the former type of sample. The main aim of this study was to identify numerical aberrations of chromosomes 7, 8, 17, X and Y in prostate cancers in Japanese men and to assess their pathological and clinical significance.

### Materials and methods

### *Sample preparation*

Fresh BPH samples were obtained from five retropubic prostatectomies and three total cystoprostatectomies. The resected prostatic tissues were cut with a disposable blade and lightly touched on precleaned slides. After air drying, the slides were incubated in 75 mM KCl for 20 min at 37°C and fixed in freshly prepared Canoy's solution (three volumes of methanol and one volume of acetic acid). They were air dried at room temperature and stored at −20°C until subsequent analysis. The touched tissues were subdivided. Portions were taken for histological examination to confirm the absence of cancerous or premalignant lesions. Among the eight BPH samples, six were frozen and stored after the initial touch preparation. Frozen-stored tissues were then slowly thawed in cold phosphate-buffered saline (PBS) (4°C), and further touch preparations were then made using the same method.

Samples from a total of 28 cases of prostatic cancer were collected, including 22 by radical prostatectomy, one by retropubic prostatectomy, three at autopsy and two from metastatic lesions (lymph node and vertebra). Twenty of the twenty-eight patients had received endocrine therapy before the excision of the tumour. The resected prostatic tissues were randomly cut at several places with disposable blades and lightly touched on precleaned slides. Samples were then stored after freezing with portions being formalin-fixed for histopathological diagnosis of paraffin-embedded sections. Only samples in which over 80% of the area was occupied by cancer and in which prostatic intraepithelial neoplasia (PIN) and adenomatous hyperplasia could be seen were used.

Correspondence: H Matsuura
Received 12 December 1995; revised 30 May 1996; accepted 28 June 1996

Frozen section was also prepared with a cryostat to confirm the presence of cancer. Cancerous tissues were thawed and touch preparations made as mentioned above. Histological classification was made according to the Gleason score method and the TNM system.

*Probes*

α-satellite DNA probes specific for centromeric regions of chromosomes 7 (D7Z1), 8 (D8Z1), 17 (D17Z1), X (DXZ1) and Y (DYZ1) were used. These probes had been labelled with digoxigenin (Oncor, Gaithersburg, MO, USA).

*Fluorescence* In situ *hybridization (FISH)*

FISH was carried out according to the manufacturer's instructions (chromosome *in situ* hybridisation system, dual colour detection for whole cells or metaphase chromosomes, fluorescence microscopy; Oncor) with slight modifications. Frozen slides were thawed at room temperature, dehydrated in ethanol and air dried. Each slide was treated with RNAase (100 $\mu g\ ml^{-1}$ in 2× standard saline citrate (SSC); Boehringer Mannheim, Germany), covered with a coverslip and placed in a moist chamber at 37°C for 20 min. Slides were rinsed in 2× SSC, dehydrated in ethanol, prewarmed at 60°C for 30 min, denatured in 70% formamide/2×SSC at 70°C for 2 min and dehydrated in cold ethanol. A probe mixture consisting of 1.5 μl of α-satellite DNA probe and 30 μl of Hybrisol VI (65% formamide/2×SSC, Oncor) per specimen was denatured at 70°C for 5 min in a water bath. The probe mixture, quickly chilled on ice for 2 min, was applied to slides which had been prewarmed to 37°C, and hybridisation was allowed to proceed overnight at 37°C in a moist chamber. The slides were post-hybridised and were washed three times at 43°C for 5 min in 65% formamide/2×SSC and two times at 37°C for 4 min in 2×SSC (pH 7.0). For detection of probes, the slides were rinsed in 3% bovine serum albumin (BSA)/0.001% Tween 20/4×SSC and incubated with anti-digoxigenin–fluorescein Fab fragments (Boehringer Mannheim) in 1% BSA/0.001% Tween 20/4×SSC at 37°C for 30 min. After three washings in 0.001% Tween 20/4×SSC at 37°C for 5 min, interphase nuclei were counterstained with propidium iodide (PI) in 90% glycol with DABCO (1,4-diazabicyclo [2.2.2.] octane; Sigma, St Louis, USA). The slides were covered with coverslips, stored at 4°C for 20 min and examined with an epifluorescence photomicroscope using an FITC and PI exciter filter cube (WIB; Olympus, Japan). The hybridising signals in 350–450 interphase nuclei were counted. In order to allow a proper evaluation of signals, the previously published criteria at Polak *et al.* (1990) were adapted. Counted nuclei were isolated and were placed so that they were not overlapping, not obstructed by thick cytoplasm and had signals with more or less the same homogeneous fluorescence intensity. Minor hybridisation spots were not counted. Spots in a paired arrangement (split spots) were counted as one signal. The percentages of nuclei which had one, two, three, four or more than five (≥5) signals per one nuclei were calculated for each specimen.

In order to obtain control values, centromere copy numbers for the five chromosomes were evaluated using eight benign prostatic tissues and the criteria described previously (Takahashi *et al.*, 1994; Brown *et al.*, 1994) with slight modifications. The mean ± s.d. for centromere copy numbers of the five chromosomes are summarised in Tables I and II. The average percentages of nuclei with two signals (disomy) for chromosomes 7, 8 and 17 were more than 91% (Figure 1a and b). The total percentages of nuclei with more than three signals (tetrasomy and other aneusomies) were quite low (<1.5%). The average percentages of nuclei with single signals of these three chromosomes were less than 6%. The average percentage of nuclei with single signals of chromosomes X and Y exceeded 93%. There was no significant difference in the distributions of centromere copy numbers between fresh and frozen-stored specimens. Cut-off values were determined based on the mean + 3 s.d. If the percentage of nuclei containing one signal was more than 12% for chromosome 7, 8 or 17, the tumour was considered as having a monosomic population. The cut-off values for three (trisomy), four (tetrasomy) and more than five (hypertetrasomy) signals for chromosome 7, 8 and 17 were 7%, 6% and 5% respectively. If the percentage of nuclei of a tumour exceeded the cut-off value at three or more points, we assumed that it had two or more cell populations with regard to numerical aberrations of chromosomes. For chromosomes X and Y, the cut-off values for two signals and ≥3 signals were 10% and 5% respectively. A tumour was considered to have a cell population without signals for chromosome Y (loss of chromosome Y) if the percentage of nuclei without any signal was more than 10% in two different trials. If the percentages of nuclei containing three and four signals simultaneously exceeded cut-off values, the more frequent one was selected as the numerical aberration. Aneuploid tumours were defined as those that had aneusomic population of at least one of the five chromosomes (modal copy number of 0, 1, 3, 5 and so forth for the centromeres of chromosome 7, 8 and 17; modal copy number of 0, 3 and so forth for the centromeres of chromosome X and Y). Tetraploidy tumours defined as those that had tetrasomic population of all five chromosomes (modal copy number of 4 for the centromeres of chromosome 7, 8 and 17; modal copy number of 2 for the centromeres of chromosome X and Y).

**Table I** Centromere copy numbers in fresh BPH samples

| *Chromosome* | Copy number[a] *1* | *2* | *3* | *4* | *≥5* |
|---|---|---|---|---|---|
| 7 | 3.7±1.3 | 93.8±2.1 | 1.5±0.9 | 0.4±0.4 | 0±0.1 |
| 8 | 4.7±1.6 | 92.4±1.6 | 1.0±0.7 | 0.4±0.2 | 0±0.3 |
| 17 | 5.7±1.8 | 91.5±1.5 | 1.1±0.7 | 0.5±0.4 | 0±0 |
| X | 96.4±1.3 | 3.1±1.0 | 0.2±0.3 | 0.1±0.2 | 0±0 |
| Y | 93.6±2.0 | 5.2±1.6 | 0.1±0.1 | 0.1±0.1 | 0±0 |

[a]Mean and standard deviation values for percentages of different centromere copy numbers for eight cases of BPH.

**Table II** Centromere copy numbers in frozen BPH samples

| *Chromosome* | Copy number[a] *1* | *2* | *3* | *4* | *≥5* |
|---|---|---|---|---|---|
| 7 | 3.7±1.7 | 93.6±1.3 | 2.0±1.1 | 0.7±0.6 | 0±0 |
| 8 | 3.7±1.9 | 93.9±1.5 | 1.8±0.9 | 0.6±0.4 | 0±0 |
| 17 | 5.1±1.7 | 92.1±2.0 | 1.9±1.0 | 0.9±0.7 | 0±0.1 |
| X | 96.3±1.1 | 3.2±1.0 | 0.9±1.2 | 0.1±0.1 | 0±0 |
| Y | 94.4±1.7 | 5.3±1.5 | 0.3±0.4 | 0±0 | 0±0 |

[a]Mean and standard deviation values for percentages of different centromere copy numbers for six cases of BPH.

*Statistical analyses*

Centromere copy numbers of interphase nuclei of fresh and frozen-stored prostatic tissue were compared using the Mann–Whitney test. Correlations between FISH analysis and pathological data were analysed with the Fisher's exact test. Statistical analyses were performed using a software program (Stat View J; Abacus Concepts, Berkeley, CA, USA) on a Macintosh Quadra 840AV computer.

### Results

*Numerical aberrations of chromosomes in prostate cancer*

Fish with α-satellite DNA probes was carried out on 28 prostatic cancers, and numerical aberrations of one or more chromosomes per tumour were detected in 20 (71%) cases (Table III). The most frequent aberration was a gain of chromosome 8, which was detected in 16 (57%) of the 28

**Figure 1** FISH with centromere-specific probes performed on touch preparation slides. (**a**) Fresh BPH specimen with nuclei showing two signals for chromosome 7. (**b**) Frozen BPH specimen with nuclei showing two signals for chromosome 7. (**c**) Frozen cancerous specimen (case 1) with nuclei showing three signals for chromosome 8. (**d**) Fresh cancerous specimen (case 28) with nuclei showing four signals and >5 signals for chromosome 7.

**Table III** Numerical aberrations of chromosomes and pathology of 28 prostate cancers

| *Case* | *Specimen*[a] | *Chromosome*[b] | | | | | *TNM* | *Gleason score* | *Ploidy pattern* |
|---|---|---|---|---|---|---|---|---|---|
| | | *7* | *8* | *17* | *X* | *Y* | | | |
| 1 | P | N | 3 | N | N | N | TxN0M0 | 7 | Aneuploid |
| 2 | P | N | 3 | N | N | N | pT2bpN0M0 | 4 | Aneuploid |
| 3 | P | 3 | 3 | N | N | N | pT2bpN0M0 | 8 | Aneuploid |
| 4 | P | N | N | N | N | N | pT2bpN0M0 | 5 | Diploid |
| 5 | P | N | N | N | N | N | pT2bpN0M0 | 4 | Diploid |
| 6 | P | N | N | N | N | N | pT3pN0M0 | 3 | Diploid |
| 7 | P | N | N | N | N | N | pT3pN0M0 | 7 | Diploid |
| 8 | P | N | N | 3 | N | N | pT3pN0M0 | 4 | Aneuploid |
| 9 | P | N | N | N | N | N | pT3pN0M0 | 3 | Diploid |
| 10 | P | N | N | 3 | N | N | pT3pN0M0 | 7 | Aneuploid |
| 11 | P | N | N | N | N | N | pT3pN0M0 | 7 | Diploid |
| 12 | P | N | 3 | N | N | N | pT3pN0M0 | 7 | Aneuploid |
| 13 | P | N | 3 | N | N | N | pT3pN0M0 | 7 | Aneuploid |
| 14 | P | N | N | N | N | N | pT3pN0M0 | 7 | Diploid |
| 15 | P | N | N | N | N | N | pT3pN1M0 | 9 | Diploid |
| 16 | P | 3 | N | N | N | N | pT2bN1M0 | 7 | Aneuploid |
| 17 | P | 4 | 3 | N | N | N | pT3pN1M0 | 7 | Aneuploid |
| 18 | P | 4 | N | N | 2 | N | pT3pN0M0 | 8 | Diploid |
| 19 | P | 4 | 4 | N | N | N | pT4pN2M0 | 8 | Diploid |
| 20 | P | 4 | 4 | 4 | 2 | N | pT2bN1M0 | 5 | Diploid |
| 21 | P | 4 | 4 | 4 | 2 | 2 | pT3pN1M0 | 8 | Tetraploid |
| 22 | P | 4,⩾5 | 4 | N | N | N | pT3pN1M0 | 7 | Aneuploid |
| 23 | P | 3,⩾5 | 3 | 4,⩾5 | N | N | pT4pN2M0 | 8 | Aneuploid |
| 24 | P | 4,⩾5 | 4,⩾5 | 4 | 2 | 2 | pT3pN2M0 | 8 | Aneuploid |
| 25 | P | 4,⩾5 | 4,⩾5 | 4,⩾5 | 2 | 2 | pT3pN1M0 | 5 | Aneuploid |
| 26 | P | 4,⩾5 | 4,⩾5 | 4 | 2 | 2 | pT3pN1M0 | 8 | Aneuploid |
| 27 | V | 3 | 3 | 3 | 2 | 0 | TxpNxM1 | 9 | Aneuploid |
| 28 | LN | 4,⩾5 | 5 | 4,⩾5 | 2,⩾3 | 0 | TxpN2M0 | 8 | Aneuploid |

[a]P, prostate; V, metastasis to vertebra; LN, metastasis to lymph node. [b]N, no numerical aberration; ⩾5, hypertetrasomy.

cancers (Figure 1c). Trisomy was the most common (8/16) with tetrasomy and hypertetrasomy being detected in 7/16 and 4/16 of the tumours respectively.

Numerical aberration of chromosome 7 was detected in 14 (50%) of the 28 tumours, trisomy and tetrasomy being found in 4 and 10 of the 28 tumours respectively. In six of these tumours, hypertetrasomy of chromosome 7 was detected together with trisomy or tetrasomy (Figure 1d).

Numerical aberrations of chromosomes 17, X and Y were detected in 11 (39%), 9 (32%) and 6 (21%) of the 28 tumours.

**Table IV** Relation between numerical aberrations of chromosomes and the total Gleason score (GS)

| *Chromosome* | *Numerical aberration (number of cases)* | *Total GS 3–7* | *8–9* | *Mean*±s.d. |
|---|---|---|---|---|
| 7 | + (14) | 5 | 9 | 7.4±1.1* |
| | − (14) | 13 | 1 | 5.8±1.9 |
| 8 | + (16) | 8 | 8 | 7.1±1.4 |
| | − (12) | 10 | 2 | 5.9±2.0 |
| 17 | + (10) | 4 | 6 | 7.0±1.7 |
| | − (18) | 14 | 4 | 6.4±1.8 |
| X | + (8) | 2 | 6 | 7.4±1.5** |
| | − (20) | 16 | 4 | 6.3±1.8 |
| Y | + (6) | 1 | 5 | 7.7±1.4** |
| | − (22) | 17 | 5 | 6.3±1.8 |
| Diploid | (11) | 8 | 3 | 6.0±2.1 |
| Aneuploid | (16) | 10 | 6 | 6.9±1.4 |

*$P<0.005$. **$P<0.05$.

**Table V** Relation between numerical aberrations and TNM stages

| *Chromosome* | *Numerical aberration (number of cases)* | *T stage pT2* | *pT3* | *pT4* | *N stage pN0* | *pN1,2* |
|---|---|---|---|---|---|---|
| 7 | + (12) | 3 | 7 | 2 | 2 | 10* |
| | − (13) | 3 | 10 | 0 | 12 | 1 |
| 8 | + (13) | 3 | 8 | 2 | 4 | 9** |
| | − (12) | 3 | 9 | 0 | 10 | 2 |
| 17 | + (8) | 1 | 6 | 1 | 2 | 6 |
| | − (17) | 5 | 11 | 1 | 12 | 5 |
| X | + (6) | 1 | 5 | 0 | 1 | 5 |
| | − (19) | 5 | 12 | 2 | 13 | 6 |
| Y | + (4) | 0 | 4 | 0 | 0 | 4** |
| | − (21) | 6 | 13 | 2 | 14 | 7 |
| Diploid | (11) | 3 | 7 | 1 | 8 | 3 |
| Aneuploid | (13) | 3 | 9 | 1 | 6 | 7 |

*$P<0.001$. **$P<0.05$.

Loss (no signal) of chromosome Y was detected in two tumours (7%). Loss of chromosomes 7, 8, 17 and X was not detected.

Twelve (75%) of the 16 cases with numerical aberrations of chromosome 8 also had changes in signals for chromosome 7. Numerical aberration of two or more chromosomes was detected in 13 cases (46%).

Using the criteria defined and described in Materials and methods, 11 diploid, one tetraploid and 16 aneuploid tumours were identified by FISH.

### *Correlation of FISH results with clinicopathological data*

Tables IV and V summarise data for relationships between numerical aberrations of chromosomes and clinicopathological features. Associations between a high Gleason score (GS) and numerical aberrations were significant for chromosomes 7 ($P<0.005$), X and Y ($P<0.05$). The 25 samples obtained at radical prostatectomy or autopsy were analysed according to the TNM system. Pathological staging was not available for the remaining three cases because histological examinations of the prostate gland and regional lymph nodes were incomplete. Numerical aberrations of individual chromosomes did not statistically correlate with T stage. Sixteen of the 19 advanced T-stage (pT3 or pT4) tumours had numerical aberrations of chromosomes 7, 8, 17, X or Y. All but one of the tumours that showed tetrasomy or hypertetrasomy were in advanced T stages. The association between lymph node metastasis and numerical aberration was most significant for chromosomes 7 ($P<0.005$), 8 and Y ($P<0.05$). The primary tumours (pN0) without lymph node metastases had a lower frequency of numerical aberrations of chromosome 7 (14%). In contrast, 88% of the pN1 tumours and all of the pN2 tumours were positive, and nine (82%) primary tumours (pN1,2) with lymph node metastasis had numerical aberrations of two or more chromosomes which included chromosome 7.

Among the cases with GS≤7, numerical aberrations of chromosome 7 were detected in all those demonstrating lymph node metastasis. All cases with tetrasomy or hypertetrasomy were primary tumours (stage D1,2) with metastasis to lymph node or other sites. Of these, 12 (86%) had numerical aberrations of two or more chromosomes.

No significant relationships were found between ploidy pattern (as determined by FISH) and GS.

Using the criteria defined and described in Materials and methods, we identified 11 diploid (39%), one tetraploid (4%) and 16 aneuploid (57%) tumours using FISH. Ploidy pattern as determined by FISH did not significantly correlate with pathological stage and GS, but aneuploidy was identified more frequently (66%) in the tumours with total GS 8 or 9. Seven (70%) of the tumours with lymph node metastases showed aneuploidy.

### Discussion

In this study, numerical aberrations were detected in 20 (71%) of 28 prostate cancers. This frequency is much higher than that found by conventional metaphase cytogenetic methods (karyotyping) (Micale *et al.*, 1992; Arps *et al.*, 1993). These methods are used after tissue culture and may result in a selective growth of cells with the highest mitotic index and loss of chromosomal material (Polak *et al.*, 1990). In contrast, FISH using chromosome-specific probes enables detection of numerical aberrations of chromosomes in interphase, as well as metaphase, cells, and the technique does not require tissue culture and can be applied to solid tumours or paraffin-embedded tissues (Micale *et al.*, 1993; Persons *et al.*, 1994). With interphase FISH, the selection that takes place in the preparation of metaphase cells from primary tumours can be avoided (Polak *et al.*, 1990). FISH is thus a useful technique for detecting numerical aberrations of chromosomes.

In this study, we used the touch preparation method, which does not require trypsin treatment and can be performed in a short time. No significant difference was found between results from preparations made with frozen-stored BPH tissues and from fresh specimens, confirming that frozen material is suitable for FISH. Similar results were obtained for the prostate cancers (data not shown).

Prostate cancers are composed of heterogeneous populations of cells with divergent Gleason scores. When the extent of cancer in the touched tissues did not exceed 80% of the total area, we used samples made from frozen-store specimens in which the presence of the cancer could be confirmed by histological examination of cryostat sections. PIN and adenomatous hyperplasia could be not seen in these touched tissues. Recently, these precursors of prostate cancer were demonstrated to have chromosomal abnormality (Alers *et al.*, 1995; Qian *et al.*, 1995*a*, *b*). Because of the advantage of the accurate sampling of cancer, FISH using frozen-stored specimens is particularly suitable for detecting the numerical aberrations of chromosomes of prostate cancers. We detected numerical aberration of each chromosome in Japanese men with prostate cancer using the criteria defined and described in Materials and methods. The cell populations with numerical aberration of any chromosome may represent a minor tumour fraction or are the result of heterogeneity of the tumour. One of the aims of this study is to correlate numerical aberration of chromosomes with clinicopathological data regardless of the cause of the aberration.

The gain of chromosome 8, the most frequent aberration detected (57%, 16 of 28) among the five chromosomes, was significantly associated with lymph node metastasis

($P<0.05$). Trisomy and hypertetrasomy were found in three quarters of the cancers. Trisomy 8 has been reported to be one of the most common aberrations in haematological malignancies (Kibbelaar *et al.*, 1991; Amiel *et al.*, 1995) analysed by FISH. The biological significance of trisomy 8 for development and progression of prostate cancer is still unclear. Genetic alterations, such as deletions or allelic loss, involving in chromosome 8 have also been recently reported (Bova *et al.*, 1993; Macosca *et al.*, 1994; Matsuyama *et al.*, 1994), suggesting the presence of putative tumour-suppressor genes. A comparison of numerical aberrations and genetic alterations of chromosome 8 would therefore be of interest.

Numerical aberration of chromosome 7 was the second most frequent anomaly in the present study of cancers (50%, 14 of 28) and was the most significantly associated with a higher GS ($P<0.005$) and with lymph node metastasis ($P<0.001$). No equivalent aberration of chromosome 7 was detected in benign prostatic specimens. Trisomy 7, which has been described as a common anomaly in solid malignant tumours (Weaver *et al.*, 1988; Belge *et al.*, 1994), was found in four (14%) cases (Case 3, 16, 23, 27). In a previous study, trisomy 7 was reported to serve as a novel marker for human prostate cancer progression (Bandyk *et al.*, 1994), and several authors (Collard *et al.*, 1987; Trent *et al.*, 1990) have suggested the presence of genes involved in metastasis and invasion on chromosome 7. Enhanced expression of epidermal growth factor receptor (EGFR) in pancreatic cancers was also reported to be associated with either structural or numerical alterations of chromosome 7 (Korc *et al.*, 1986). In addition, polysomy of chromosome 7 has been linked with overexpression and amplification of the EGF-receptor gene in human carcinoma cell lines (Helseth *et al.*, 1990). Our findings suggest that a gain of chromosome 7 in prostate cancer may play an important role in progression and especially metastasis, and that this may be associated with aggressive tumour behaviour and a poor prognosis in patients with prostate cancer.

Aberrations in chromosome 17, X and Y, except two cases with trisomy 17, accompanied numerical aberrations of chromosome 7 or 8 and were detected in tumours with high GS, lymph node metastasis or metastatic sites. These findings suggest that multiple chromosomal alterations, including those of chromosomes 7 or 8, are common in aggressive prostate cancer. In contrast to the low incidence found here (4%), loss of chromosome Y has been reported to be frequent in prostate cancers in western countries. European reports demonstrated that loss of Y was detected by FISH in 53% (Konig *et al.*, 1994) and in 8 of 26 (31%) primary prostatic adenocarcinomas (Breitkreuz *et al.*, 1993). In Americans, cytogenetic studies after short-term culture showed that the most frequent numerical changes included loss of chromosome Y in 4 of 32 (13%) adenocarcinomas of the prostate from patients without prior treatment (Arps *et al.*, 1993), and that the most common clonal numerical aberration was loss of Y in 5 of 62 (8%) prostatic adenocarcinomas (Micale *et al.*, 1992). A previous report (Aly *et al.*, 1994) combining conventional cytogenetic analysis and FISH of short-term culture of benign prostatic hyperplasia showed loss of chromosome Y to be the most common chromosomal change. The cause of our low frequency might be the result of technical variation.

Interphase cytogenetics is suitable for detecting gain of chromosomes but it can be difficult to distinguish focal-insufficient hybridisation from chromosomal loss (Wolman *et al.*, 1992). We diagnosed loss of chromosome X and Y only when nuclei with perfectly hybridising signals and nuclei without any signal were detected in the same sample in two different trials, as recommended in a previous study (Henke *et al.* 1994*a*). One report demonstrated that loss of chromosome Y by FISH was noted in fewer cases (10%) than would be expected from the literature (Henke *et al.*, 1994*b*). This policy might have resulted in the low frequency of loss of sex chromosomes. An alternative explanation for the discrepancy is the difference in material. Thus, genetic changes in prostate cancer have been reported to differ between Japanese and American men. The frequency of *ras* mutations varies according to ethnic groups (Watanabe *et al.*, 1994*a*) and the *p53* mutational spectrum in Japanese prostate cancer patients is different from that in American prostate cancer patients (Watanabe *et al.*, 1994*b*). To clarify these questions, we need to analyse the materials collected from different ethnic groups with the same analytical procedures.

In conclusion, the present study showed that FISH analysis of touch preparation slides from fresh or frozen-stored specimens is a useful method for detecting numerical aberration of individual chromosomes. In our series of prostate cancers in Japanese men, numerical aberration of chromosome 8 was the most common phenomenon, while changes in chromosome 7 were suggested to have potential as markers of aggressive tumour behaviour and a poor prognosis. In addition, multiple numerical aberrations involving several chromosomes, including chromosome 7 and/or 8, proved common in aggressive prostate cancers. The association between genetic change and chromosomal abnormality should be studied in detail. Our data on chromosomal aberrations in Japanese prostate cancers except those on the loss of chromosome Y are consistent with those reported in Western countries.

### References

ALERS JC, KRIJTENBURG PJ, VISSERS KJ, BOSMAN FT, VAN DER KWAST TH AND VAN DEKKEN H. (1995). Interphase cytogenetics of prostatic adenocarcinoma and precursor lesions: analysis of 25 radical prostatectomies and 17 adjacent prostatic intraepithelial neoplasias. *Genes Chrom. Cancer*, **12**, 241–250.

ALY MS, CIN PD, VAN DE VOORDE W, VAN POPPEL H, AMEYE F, BAERT L AND VAN DEN BERGHE H. (1994). Chromosome abnormalities in benign prostatic hyperplasia. *Genes Chrom. Cancer*, **9**, 227–233.

AMIEL A, GABER E, MANOR Y, FEJGIN M, JOSEEPH-LERNER N, RAVID M AND LISHNER M. (1995). Fluorescence in situ hybridization for the detection of trisomies 8 and 9 in polycythemia vera. *Cancer Genet. Cytogenet.*, **79**, 153–156.

ARPS R, RODEWALD A, SCHMALENBERGER B, CARL P, BRESSEL M AND KASTENDIECK H. (1993). Cytogenetic survey of 32 cancers of the prostate. *Cancer Genet. Cytogenet.*, **66**, 93–99.

BANDYK MG, ZHAO L, TRONCOSO P, PISTERS LL, PALMER JL, VON ESCHENBAC AC, CHUMG LW AND LIANG JC. (1994). Trisomy 7: a potential cytogenetic marker of human prostate cancer progression. *Genes Chrom. Cancer*, **9**, 19–27.

BELGE G, THOBE B, RIPPE V, BARTNITZEKE S AND BULLERDIEK J. (1994). A characteristic sequence of trisomies starting with trisomy 7 in benign thyroid tumors. *Hum. Genet.*, **94**, 198–202.

BOVA GS, CARTER BS, BUSSEMAKERS MJ, EMI M, FUJIWARA Y, KYPRIANOU N, JACOBS SC, ROBINSON JC, EPSTEIN JI AND WALSH PC. (1993). Homozygous deletion and frequent allelic loss of chromosome 8p22 loci in human prostate cancer. *Cancer Res.*, **53**, 3869–3873.

BREITKREUZ T, ROMANAKIS K, LUTZ S, SEITZ G, BONKHOFF H, UNTEREGGER G, ZWERGEL T, ZANG KD AND WULLICH B. (1993). Genotypic characterization of prostatic carcinomas: a combined cytogenetic, flow cytometry, and in situ DNA hybridization study. *Cancer Res.*, **53**, 4035–4040.

BROTHMAN AR, PEEHL DM, PATEL AM AND MCNEAL JE. (1990). Frequency and pattern of karyotypic abnormalities in human prostate cancer. *Cancer Res.*, **50**, 3795–3803.

BROTHMAN AR, WATSON MJ, ZHU XL, WILLIAMS BJ AND ROHR LR. (1994). Evaluation of 20 archival prostate tumor specimens by fluorescence in situ hybridization (FISH). *Cancer Genet. Cytogenet.*, **75**, 40–44.

BROWN JA, ALCARAZ A, TAKAHASHI S, PERSONS DL, LIEBER MM AND JENKINS RB. (1994). Chromosomal aneusomies detected by fluorescent in situ hybridization analysis in clinically localized prostate carcinoma. *J. Urol.*, **152**, 1157–1162.

Collard JG, van de Poll M, Scheffer A, Roos E, Hopman AH, Geurts van Kessel AH and van Dongen JJ. (1987). Location of genes involved in invasion and metastasis on human chromosome 7. *Cancer Res.*, **47**, 6666–6670.

Cremer T, Landegent J, Bruckner A, Scholl H, Schardin M, Hager H, Devilee P, Pearson P and van der Ploeg M. (1986). Detection of chromosome aberrations in human interphase nucleus by visualization of specific target DNAs. *Hum. Genet.*, **74**, 346–352.

deVilee P, Thirry RF, Kievits T, Kolluri R, Hopman AH, Willard HF, Pearson PL and Cornelisse CJ. (1988). Detection of chromosome aneuploidy in interphase nuclei from human primary breast tumors using chromosome-specific repetitive DNA probes. *Cancer Res.*, **48**, 5825–5830.

Helseth E, Brogger A, Dalen A, Fure H, Johansen SG, Lier ME, Skandsen T, Unsgaard G and Vik R. (1990). Polysomy of chromosome 7 is associated with amplification and overexpression of the EGF-receptor gene in a human carcinoma cell line derived from a brain metastasis. *Acta Pathol. Microbiol. Immunol. Scand.*, **98**, 996–1004.

Henke RP and Ayhan N. (1994*a*). Enhancement of hybridization efficiency in interphase cytogenetics on paraffin-embedded tissue sections by microwave treatment. *Anal. Cellular Pathol.*, **6**, 319–325.

Henke RP, Kruger E, Ayhan N, Hubner D and Hammerer P. (1994*b*). Frequency and distribution of numerical chromosomal aberrations in prostatic cancer. *Hum. Pathol.*, **25**, 476–484.

Hopman AHN, Ramaekers FCS and Vooijs GP. (1990). Interphase cytogenetic of solid tumor, In *In Situ Hybridization, Principles and Practice.*, Polak JM and McGee JO (eds) pp.165–186. Oxford University Press: Oxford.

Kibbelaar RE, van Kamp H, Dreef EJ, Wessels JW, Beverstock GC, Raap AK, Fibbe WE, den Ottolander GL and Kluin PM. (1991). Detection of trisomy 8 in hematological disorders by in situ hybridization. *Cytogenet. Cell Genet.*, **56**, 132–136.

Konig JJ, Teubel W, van Dongen JW, Romijn JC, Hagemeijer A and Schronder FH. (1994). Loss and gain of chromosomes 1, 18, and Y in prostate cancer. *Prostate*, **25**, 281–291.

Korc M, Meltzer P and Trent J. (1986). Enhanced expression of epidermal growth factor receptor correlates with alterations of chromosome 7 in human pancreatic cancer. *Proc. Natl Acad. Sci. USA*, **83**, 5141–5144.

Macosca JA, Trybus TM, Sakr WA, Wolf MC, Benson PD, Powell IJ and Pontes JE. (1994). Fluorescence in situ hybridization analysis of 8p allelic loss and chromosome 8 instability in human prostate cancer. *Cancer Res.*, **54**, 3824–3830.

Matsuyama H, Pan Y, Skoog L, Tribukait B, Naito K, Ekman P, Lichter P and Bergerheim US. (1994). Deletion mapping of chromosome 8p in prostate cancer by fluorescence in situ hybridization. *Oncogene*, **9**, 3071–3076.

Micale MA, Mohammed A, Sakr W, Powell IJ and Wolman SR. (1992). Cytogenetics of primary adenocarcinoma: clonality and chromosome instability. *Cancer Genet. Cytogenet.*, **61**, 165–173.

Micale MA, Sanford JS, Powell IJ, Sakr WA and Wolman SR. (1993). Defining the extent and nature of cytogenetic events in prostatic adenocarcinoma: paraffin FISH vs. metaphase analysis. *Cancer Genet. Cytogenet.*, **69**, 7–12.

Persons DL, Hartmann LC, Herath JF, Borell TJ, Cliby WA, Keeney GL and Jenkins RB. (1993). Interphase molecular cytogenetic analysis of epithelial ovarian carcinomas. *Am. J. Pathol.* **142**, 733–741

Persons DL, Takai K, Gibney DJ, Katzmann JA, Lieber MM and Jenkins RB. (1994). Comparison of fluorescence in situ hybridization with flow cytometry and static image analysis in ploidy analysis of paraffin-embedded prostate adenocarcinoma. *Hum. Pathol.*, **25**, 678–683.

Pinkel D, Straume T and Gray JW. (1986). Cytogenetic analysis using quantitative, high-sensitivity, fluorescence hybridization. *Proc. Natl Acad. Sci. USA*, **83**, 3934–3938.

Qian J, Bostwick DG, Takahashi S, Borell TJ, Herath JF, Lieber MM and Jenkins RB. (1995*a*). Chromosomal anomalies in prostatic intraepithelial neoplasia and carcinoma detected by fluorescence in situ hybridization. *Cancer Res.*, **55**, 5408–5414.

Qian J, Jenkins RB and Bostwick DG. (1995*b*). Chromosomal anomalies in atypical adenomatous hyperplasia and carcinoma of the prostate using fluorescence in situ hybridization. *Urology*, **46**, 837–842.

Takahashi S, Qian J, Brown JA, Alacaraz A, Bostwick DG, Lieber MM and Jenkins RB. (1994). Potential markers of prostate cancer aggressiveness detected by fluorescence in situ hybridization in needle biopsies. *Cancer Res.*, **54**, 3574–3579.

Trask B and Pinkel D. (1990). Fluorescence in situ hybridization with DNA probe. *Methods Cell Biol.*, **33**, 383–400.

Trent JM, Meysken FL, Salman SE, Ryschon K, Leong SP, Davis JR and McGee DL. (1990). Relation of cytogenetic abnormalities and clinical outcome in metastatic melanoma. *N. Engl. J. Med.*, **322**, 1508–1511.

Visakorpi T, Hyytinen E, Kallioniemi A, Isola J and Kallioniemi OP. (1994). Sensitive detection of chromosome copy number aberrations in prostate cancer by fluorescence in situ hybridization. *Am. J. Pathol.*, **145**, 624–630.

Waldman FM, Carroll PR, Kerschmann R, Cohen MB, Field FG and Mayall BH. (1991). Centromeric copy number of chromosome 7 is strongly correlated with tumor grade and labeling index in human bladder cancer. *Cancer Res.*, **51**, 3807–3813.

Watanabe M, Shiraishi T, Yatani R, Nomura AM and Stemmermann GN. (1994*a*). International comparison on ras gene mutations in latent prostate carcinoma. *Int. J. Cancer*, **58**, 174–178.

Watanabe M, Ushijima T, Kakiuchi H, Shiraishi T, Yatani R, Shimazaki J, Kotake T, Sugimura T and Nagao M. (1994*b*). p53 gene mutations in human prostate cancers in Japan: different mutation spectra between Japan and western countries. *Jpn J. Cancer Res.*, **85**, 904–910.

Weaver DJ, Michalski K and Miles K. (1988). Cytogenetic analysis in renal cell carcinoma: correlation with tumor aggressiveness. *Cancer Res.*, **48**, 2887–2889.

Wolman SR, Macoska JA, Micale MA and Sakr WA. (1992). An approach to definition of genetic alterations in prostate cancer. *Diag. Molecular Pathol.*, **1**, 192–199.

Zitzelsberger H, Szucs S, Weier HU, Lehmann L, Braselmann H, Ender S, Schilling A, Breul J, Hofler H and Bauchinger. (1994). Numerical abnormalities of chromosome 7 in human prostate cancer detected by fluorescence in situ hybridization (FISH) on paraffin-embedded tissue sections with centromere-specific DNA probes. *J. Pathol.*, **172**, 325–335.